# Diversity of short linear interaction motifs in SARS-CoV-2 nucleocapsid protein

**DOI:** 10.1128/mbio.02388-23

**Published:** 2023-11-29

**Authors:** Peter Schuck, Huaying Zhao

**Affiliations:** 1 Laboratory of Dynamics of Macromolecular Assembly, National Institute of Biomedical Imaging and Bioengineering, National Institutes of Health, Bethesda, Maryland, USA; National Institutes of Health, Bethesda, Maryland, USA

**Keywords:** virus-host interactions, short linear motifs, quasispecies, intrinsically disordered protein domains

## Abstract

Molecular mimicry of short linear interaction motifs has emerged as a key mechanism for viral proteins binding host domains and hijacking host cell processes. Here, we examine the role of RNA virus sequence diversity in the dynamics of the virus-host interface by analyzing the uniquely vast sequence record of viable SARS-CoV-2 species with focus on the multi-functional nucleocapsid protein. We observe the abundant presentation of motifs encoding several essential host protein interactions, alongside a majority of possibly non-functional and randomly occurring motif sequences absent in subsets of viable virus species. A large number of motifs emerge *ex nihilo* through transient mutations relative to the ancestral consensus sequence. The observed mutational landscape implies an accessible motif space that spans at least 25% of known eukaryotic motifs. This reveals motif mimicry as a highly dynamic process with the capacity to broadly explore host motifs, allowing the virus to rapidly evolve the virus-host interface.

**IMPORTANCE:**

Short linear motifs (SLiMs) are 3–10 amino acid long binding motifs in intrinsically disordered protein regions (IDRs) that serve as ubiquitous protein-protein interaction modules in eukaryotic cells. Through molecular mimicry, viruses hijack these sequence motifs to control host cellular processes. It is thought that the small size of SLiMs and the high mutation frequencies of viral IDRs allow rapid host adaptation. However, a salient characteristic of RNA viruses, due to high replication errors, is their obligate existence as mutant swarms. Taking advantage of the uniquely large genomic database of SARS-CoV-2, here, we analyze the role of sequence diversity in the presentation of SLiMs, focusing on the highly abundant, multi-functional nucleocapsid protein. We find that motif mimicry is a highly dynamic process that produces an abundance of motifs transiently present in subsets of mutant species. This diversity allows the virus to efficiently explore eukaryotic motifs and evolve the host-virus interface.

## INTRODUCTION

Short linear interaction motifs (SLiMs) are stretches of several amino acids that serve as microdomains in intrinsically disordered regions (IDRs) mediating weak, transient protein-protein interactions with target domains (1–3). SLiMs have emerged as ubiquitous modules in the organization of protein-protein interaction networks. For example, SLiMs designate substrates for post-translational modification including kinases and phosphatases; target cellular localization; and control docking to adaptors, assembly of signaling complexes, and recruitment of enzymes to multi-protein complexes (2, 4–10). The number of eukaryotic SLiM classes is in the hundreds and rapidly expanding, and it has been estimated that there may be more than a hundred thousand instances of such motifs in the human proteome (3, 11, 12).

Molecular mimicry of SLiMs is a key mechanism for viral proteins to hijack and modulate host cell processes and is broadly exploited among many viruses (13–15). Therefore, the distribution and evolution of viral motifs are of significant interest in the search for broad-spectrum anti-viral drug targets (16, 17). It has been proposed that the evolution of SLiMs is facilitated by their compact size, in combination with the unusually high abundance of IDRs in viral proteins; the latter, by virtue of fewer constraints, generally exhibiting high mutation frequencies that would allow the efficient *de novo* formation of SLiMs through as little as a single amino acid change (18–23). The conservation of disorder has been hypothesized to facilitate change of interaction partners and thereby provide an evolutionary advantage (24). In eukaryotes, the generation and loss of SLiMs have been confirmed in sequence analyses of evolutionarily related species as well as in rare random mutations in individual human patients (21, 25). In viruses, substantial heterogeneity of motif content has been observed across different viral families, with instances of convergent evolution, pointing to a highly dynamic repertoire of motif usage and evolutionary adaptation (13).

On the other hand, a salient feature of RNA viruses is their high intracellular and intrahost sequence diversity due to their evolved low transcription fidelity and resulting quasispecies nature (26–29). How this sequence diversity impacts the virus-host interface and the evolution of SLiMs has remained unexplored. An opportunity to study this question recently arose with the unprecedented, vast collection SARS-CoV-2 genomes in GISAID (30). While it provides a basis for monitoring the evolution of mutations distinguishing emergent clades, the repository contains a majority of random transient mutations that exhaustively explore the mutational landscape of viral proteins (31–33). In the present work, we exploit the diversity of SARS-CoV-2 sequences and examine the distribution of viral motif content on the infected host population level, which we propose may serve as a model also for intrahost viral motif diversity.

**Fig 1:**
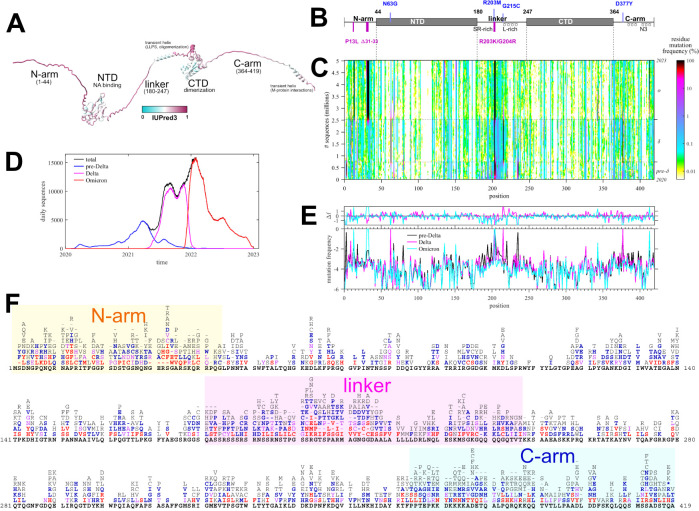
Mutational landscape of N-protein. (**A**) Predicted AlphaFold structure of N-protein, with extended IDRs for clarity, colored according to the disorder score. Labeled are the folded domains (NTD, CTD) and the three IDRs (N-arm, linker, and C-arm) with known assembly functions. (**B**) Schematic organization of N-protein with defining mutations of Delta (clade 21J, blue) and Omicron variant (magenta). (**C**) Time-dependent mutation frequencies (colors) at different positions (abscissa) plotted as a function of cumulative sequences (ordinate). The dotted horizontal lines are the time-points where Delta and Omicron variants rose to majority, as indicated in the history of daily deposited sequence numbers grouped by pre-Delta (comprising ancestral, Alpha, Beta, and other variants predating Delta 21J), Delta, and Omicron variants (**D**). (**E**) Average mutation frequencies of residues along N-protein positions, in log10 units, grouped by major variants as in panel** D**. The upper panel shows the difference in average mutation frequencies of Delta and Omicron relative to the pre-Delta group. (**F**) Detailed list of observed amino acid mutations in each position, colored by the number of observed instances (≥1,000 red, ≥500 magenta, ≥100 blue, ≥10 gray, ancestral black).

Eukaryotic motif mimicry for cell entry has been found in the receptor-binding domain of the spike protein of SARS-CoV-2 (34). In the present work, we focus on the nucleocapsid (N-)protein, which has several favorable properties as platform for SLiMs, having the highest expression level of all SARS-CoV-2 proteins at ≈1% of total protein in infected cells (35) and containing three IDRs spanning nearly half of the protein (Fig. 1A). Even though it provides a major antigen, it is not immuno-dominant as the spike protein. Besides its eponymous structural role in viral assembly (36–38), N-protein is highly multi-functional with a large host interactome (39–42), including interactions with proteins of the type 1 interferon signaling pathway (43–45), the inflammasome (46), complement activation (47), lipid metabolism (48), and expression and binding to cytokines (49, 50). Among interactions described in greatest biophysical detail are the complex formation with G3BP1 leading to rewiring of stress granules (40, 51, 52), binding of 14-3-3 (35, 53, 54), and the interaction with host kinases leading to extensive phosphorylation particularly in the linker IDR of intracellular N-protein primed by SR-rich splicing factor protein kinase (SRPK) (55–57). Other posttranslational modifications include ubiquination, proteolytic cleavage, sumoylation, and ADP-ribosylation (58–60).

The basis for the present work is a data set of ≈5 million SARS-CoV-2 consensus sequences, which we first characterize with respect to the mutation frequencies and mutational landscape of N-protein over time. We have recently exploited the exhaustive map of viable amino acid mutations as a tool in the structural analysis of assembly roles of the linker IDR (31, 36). For the analysis of motif content, we examine the ancestral consensus sequence and utilize a previously introduced statistical approach (13) to identify possibly random motif instances. We then determine the distribution of motif content in the ensemble of viral sequences sampled across the infected host population. This analysis reveals a highly dynamic motif presentation where most of the ancestral motifs are abandoned in at least some sequence subsets, while in others, large numbers of new motifs arise that potentially interact with various host pathways. Finally, based on the mutational landscape, we estimate a measure of the available sequence space and corresponding accessible motif space, which suggests that even for the smallest N-protein IDR, a large fraction of known motifs may be presented. While many or most of these may not be functionally interacting with host proteins, it reveals a mechanism for extensive probing of viral proteins for potentially beneficial host protein interactions. We discuss possible implications for the evolution of the virus-host interface considering intrahost and intracellular sequence diversity.

## RESULTS

### The mutational landscape of SARS-CoV-2 nucleocapsid protein

The present study is based on 5.06 million high-quality consensus SARS-CoV-2 sequences retrieved on 20 January 2023 from Nextstrain (61). Due to their origin from COVID-19 patient samples, we may assume these to be sequences of viable and infectious virus. The sequences exhibit significant diversity, with ≈56% of sequences being distinct, ≈30% unique, and ≈8% spatio-temporally distant repeats. The set contains ≈43 million instances of N-protein mutations relative to the ancestral Wuhan-Hu-1 isolate. Most of the mutations are different from the defining substitutions of the variants of concern (Fig. 1B) and instead occur transiently and are distributed across ≈92% of all N-protein residues. This suggests that these mutations may have a significant impact on the N-protein motif repertoire.

Figure 1C shows the frequency of these evolutionarily seemingly inconsequential mutations at different positions and as a function of time (where time is plotted in the ordinate transformed to a cumulative sequence number to compensate for uneven sampling frequency [Fig. 1D]). As may be discerned from the barcode-like vertical patterns in the frequency plot Fig. 1C, the local mutation frequencies are in first approximation constant outside the defining variant substitutions, with minor evidence of limited stochastic transmission events causing temporal variation. By contrast, there is significant structure across different positions: higher frequencies are generally observed in the IDRs, and lower mutation frequencies correlate with biophysical functional constraints in the folded domains and IDRs (31, 36). Short of a detailed evolutionary analysis, we may roughly group sequences into three distinct sets of Omicron variants, Delta variants, and those preceding the Delta variants, which constitute the vast majority of sequences in different periods of time (Fig. 1D). Their residue mutation frequency pattern is nearly identical outside the defining substitutions, which shows that major constraining biophysical properties of N-protein are unaltered. This is highlighted further in the detailed comparison of average residue mutation frequencies as a function of position among the three groups (Fig. 1E).

A detailed chart of the observed amino acid mutations at all positions is shown in Fig. 1F. On average, ≈5.5 different amino acids may occupy each position, ranging from zero mutations at 14 of the 37 conserved positions across related coronaviruses, to a maximum of 12 different amino acids that may occupy the most variable positions of the IDRs. Their pattern defines a characteristic mutational landscape, which is similar to that reported previously (31), despite the fact that the current data set includes ≈2.6 million Omicron sequences (separately shown in Fig. S1) that were not yet available previously. We conclude that the different waves of SARS-CoV-2 variants independently reproduce very similar mutational landscapes, as would be expected for exhaustively sampled N-protein in a steady state.

The mutational landscape is a comprehensive set of all tolerable, non-lethal mutations (62), and as such, it reflects detailed biophysical constraints and provides complementary information to traditional structural tools (31, 36). For example, binding to G3BP1/2 was identified as an essential N-protein function, and a crystal structure shows G3BP1 binding the ϕ-x-F motif in the N-arm (51). Accordingly, F17 is an almost completely conserved residue in the mutational landscape of the otherwise highly variable IDR (Fig. 1F). Similarly, in the leucine-rich region of the linker IDR, the oligomerization of transient helices to form coiled coils was recently identified as an essential assembly function, and structural requirements for oligomerization were found to be reflected in the nature of the limited set of amino acid mutations in positions 221–233, in the otherwise highly variable linker IDR (36).

Different combinations of N-protein mutations define 40,988 distinct N-protein sequences. Since linear motifs are prevalent in IDRs, we focus on the subset of distinct IDR sequences. Using a threshold condition that each sequence is observed in at least 10 different genomes, among the variant groups there are a total of 611 distinct sequences for N-arm carrying an average of 2.75 mutations, 1045 for the linker IDR with an average of 2.90 mutations, and 685 for the C-arm IDR with an average of 1.77 mutations. Each sequence was examined with regard to their SLiM content using the Eukaryotic Linear Motif (ELM) Resource for Functional Sites in Proteins (11), which can search for occurrences of regular expressions of 327 documented motif classes.

### Motif content of the ancestral SARS-CoV-2 N-protein sequence

**Fig 2:**
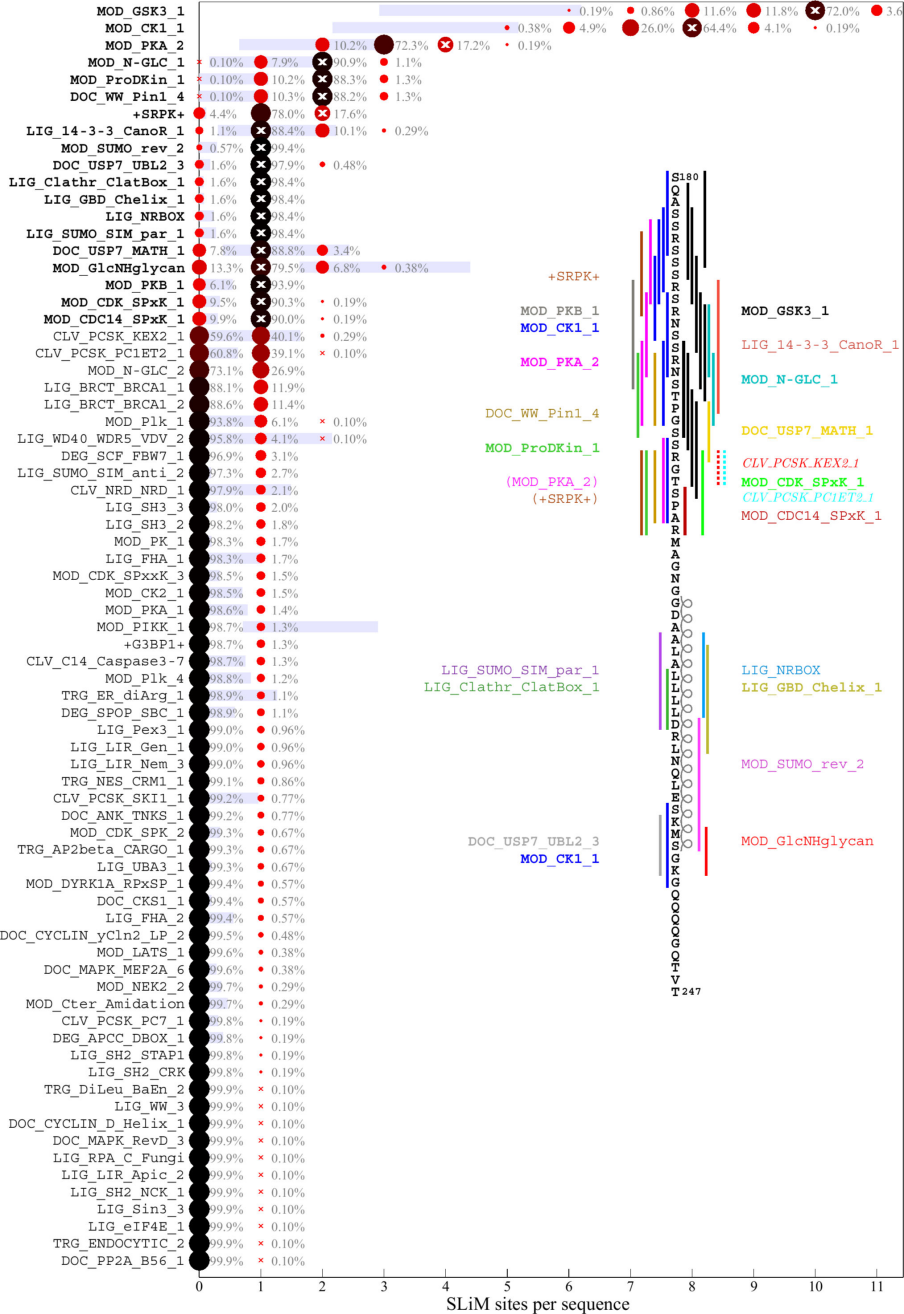
Predicted SLiM diversity in the linker IDR. Each row presents a histogram for the number of sites (abscissa) of a motif class in the ancestral reference sequence (bold motif name) or in any of the 1045 distinct mutant linker sequences (normal font motif name). The frequency of the site numbers across the ensemble of sequences is indicated by symbol size and color, as well as the listed percentage value. The motif site number in the ancestral sequence is indicated by a white cross. The blue bars represent the mean ± standard deviation of the abundance of each motif in 10,000 randomly permutated reference sequences. The inset shows the location of the ancestral motifs as vertical bars and correspondingly colored motif name. New motifs emerging in the Omicron variant due to the defining R203K/G204R mutation are indicated as dotted lines and italicized motif name, and disappearing motifs are indicated in parenthesis.

**Fig 3:**
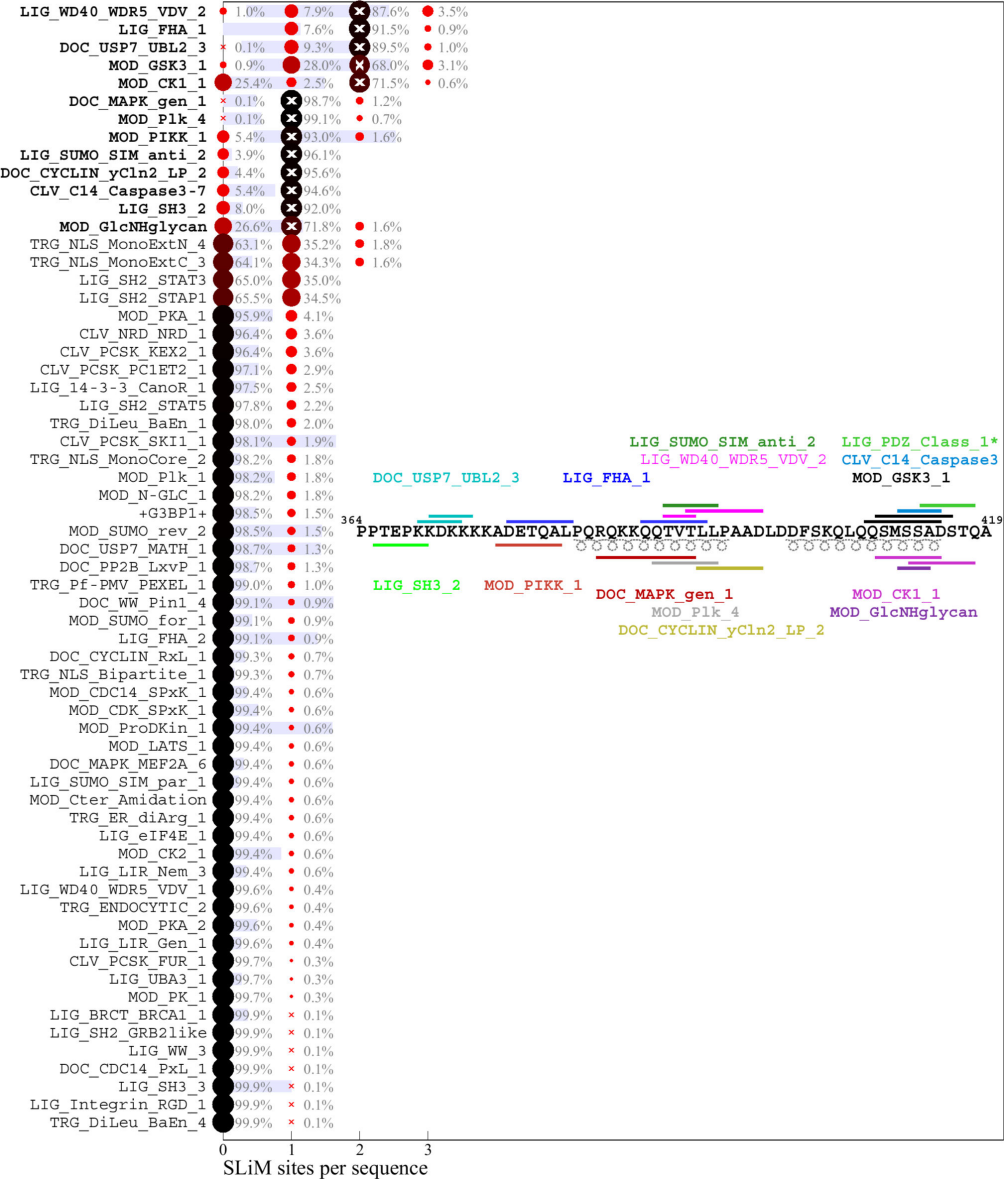
Predicted SLiM diversity in the C-arm IDR. Histograms for the number of sites of motif classes in the ancestral reference sequence (bold) or in any of the 685 distinct mutant C-arm sequences. Symbols and labels are as in Fig. 2. The inset shows the location of the ancestral motifs as horizontal bars and correspondingly colored motif name. *LIG_PDZ_Class_1 is excluded from the distribution analysis (see Materials and Methods).

**Fig 4:**
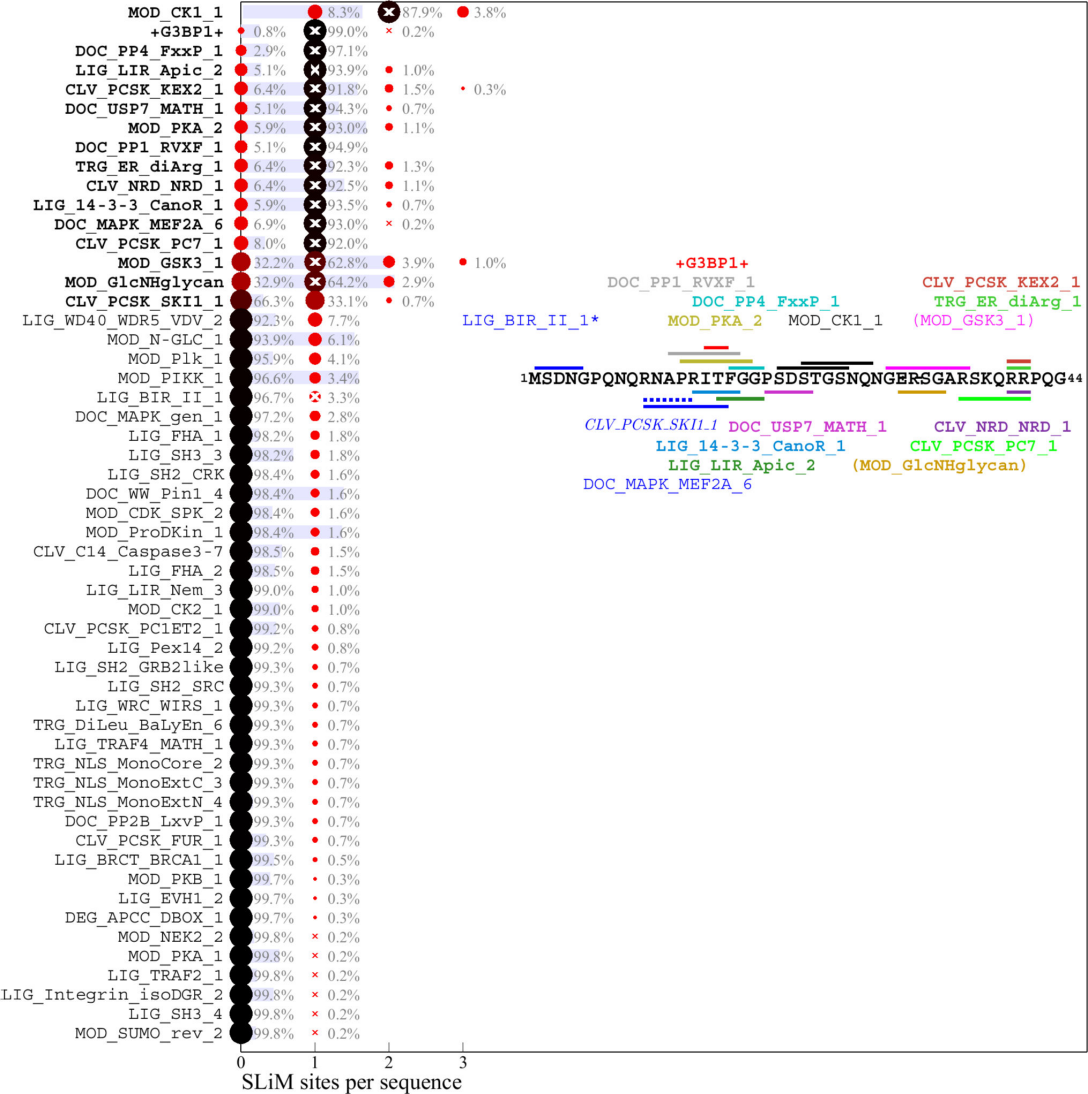
Predicted SLiM diversity in the N-arm IDR. Histograms for the number of sites of motif classes in the ancestral reference sequence (bold) or in any of the 611 distinct mutant N-arm sequences. Symbols and labels are as in Fig. 2. The inset shows the location of the ancestral motifs as horizontal bars and correspondingly colored motif name. Omicron sequences have a defining substitution P13L and deletion in positions 31–33. As a consequence, motifs in parenthesis do not occur in Omicron sequences, and motifs written in italics emerged. *LIG_BIR_II_1 is excluded from the distribution analysis (see Materials and Methods).

As a starting point to parse the results, we consider first the motif content in the linker IDR of the ancestral Wuhan-Hu-1 sequence. Figure 2 lists in bold the predicted ancestral motifs, and the inset shows their location. Many motif classes occur in multiple instances; their number charted as white crosses. The motif set is dominated by sites for kinases, in particular, in the SR-rich region. This is not surprising, considering the high degree of phosphorylation experimentally observed (55, 63). Kinase motifs significantly overlap, which may produce allovalency and allow for cooperativity and increased effective affinity of the sites for kinase binding (64). In addition, several other motifs overlap in both the SR-rich and the transiently helical L-rich region of the linker (Fig. 1B). Such overlap may not preclude their independent function considering the large number of intracellular copies of N-protein. A previously reported 14-3-3 motif in the linker (53, 54) is reproduced, and a variety of motifs for different posttranslational modifications and binding functions are found. A similar preponderance of phosphorylation motifs is found in the C-arm (Fig. 3) and N-arm (Fig. 4) IDRs. While some of the other motifs for protein modification and host protein interactions seem plausible, such as those related to de-ubiquination, sumoylation, autophagy, and apoptosis, others appear unlikely to describe real interactions, for example, several glycosylation motifs (65) and those targeting proteins of different organisms.

As a measure for the likelihood that some of these motifs may appear just stochastically, we employ a strategy previously developed by Hagai and co-workers (13): for each of the IDRs, we generated a set of 10,000 randomly scrambled sequences with the same amino acid content, and for each motif, we determined the frequency of it occurring in the randomized set. The average number of sites with standard deviation is depicted as blue bars in Fig. 2 to 4. As shown in the first three rows of Fig. 2, there is a relatively high probability of generating multiple GSK3, CK1, and PKA phosphorylation sites in the linker IDR by chance, which may be expected given the amino acid composition particularly of the SR-rich region (Fig. 2). However, their actual number in the ancestral sequence (white crosses) exceeds the statistical expectation, consistent with the important role of phosphorylation for intracellular N-protein (55, 56). Other motifs of the ancestral sequence that have a high probability to occur by chance given the linker amino acid composition are sites for binding of 14-3-3 protein, USP7, and glycosaminoglycan attachment. Interestingly, a motif for interaction with yeast KEX2 protease has a high statistical chance to occur and indeed is created through the defining R203K/G204R mutation of Alpha, Gamma, and Omicron variants. In the C-arm and N-arm IDRs, the majority of motifs displayed in the ancestral sequence are also likely to occur by chance given the respective IDR amino acid compositions. Unfortunately, the statistical analysis breaks down for motifs utilizing amino acids that are not part of the ancestral sequence, such as the defining 215C mutation of Delta variant linker, which not only enhances assembly functions (31) but also creates a likely non-functional N-glycosylation motif.

### Distribution of motifs in the mutant spectrum of SARS-CoV-2 N-protein

The distribution of motifs across the observed sequences can be depicted as a histogram of the site multiplicity. Accordingly, for each motif in Fig. 2 to 4, the color and size of the circles are scaled from large black to small red according to the frequency of sequences exhibiting different numbers of instances of that motif, as indicated numerically. A complete list of SLiMs and their frequencies can be downloaded from https://doi.org/10.7910/DVN/HOKVVE. Strikingly, the major phosphorylation motifs in the linker (Fig. 2) exhibit a great polydispersity in site numbers, for example, ranging from 6 to 11 with a mode of 10 for GSK3, from 5 to 10 with a mode of 8 for CK1, and from 2 to 5 with a mode of 3 for PKA sites. The sum of phosphorylation motifs in the SR-rich region of the linker IDR ranges from 16 to 29. Even though a higher than statistically expected number of phosphorylation motifs is conserved across all sequences, it appears as if the detailed phosphorylation events are not critical for viable virus, as judged by the fact that individually, none of the phosphorylation sites in the SR-rich region are conserved in the mutational landscape (Fig. 1F). Interestingly, most of the sequences have one less predicted PKA site and one less SRPK site than the ancestral sequence, which is caused by defining mutations R203K/G204R in Alpha, Gamma, and Omicron, and the R203M mutation in Delta variant; these mutations have been shown experimentally to cause reduced phosphorylation and enhanced assembly and replication functions and were hypothesized to reflect viral evolution (55, 57). A similar picture of prominent polydispersity emerges in the motif distributions of the C-arm and N-arm IDRs (Fig. 3 and 4).

Regarding the likelihood of motifs occurring by chance given the amino acid composition of the linker IDR, it is interesting to note that motifs with high statistical chance are indeed found in greater numbers in sequence subsets, which may be discerned for the glycosaminoglycan attachment, 14-3-3 binding, and USP7 binding motifs in the linker, as well as the GSK3 binding and glycosaminoglycan attachment sites in the N-arm.

A striking aspect of the motif content in the mutant spectrum is that nearly all motifs appear dispensable, which is indicated in the distinct sequence populations with zero sites in Fig. 2 to 4. The only exceptions are phosphorylation motifs of the linker and N-arm, and the FHA binding motif in the C-arm IDR. All other motifs are absent in sometimes sizeable fractions of mutant virus sequences, ostensibly suggesting that they may not describe real host protein interactions or that these are not an essential part of the virus-host interface (see Discussion). For example, 1.1% of all linker sequences lack the 14-3-3 site, and 9.9% lack the CDC14 phosphatase dephosphorylation site, 25.4% of C-arm sequences lack both CK1 phosphorylation sites, and 32.2% of N-arm sequences lack the GSK3 site. Similarly, the defining Δ31-33 mutation in Omicron sequences destroys a likely non-functional glycosaminoglycan motif.

Conversely, many motifs that do not exist in the ancestral sequence are formed *ex nihilo* in subsets of sequences due to their particular constellation of mutations. In addition to several motifs arising from defining mutations of variants of concern (such as the yeast KEX2 protease site mentioned above), a large number of motifs occur in only a small fraction of sequences. However, since only sequences occurring in at least 10 different genomes were included in the analysis, a frequency of ≈1% of all linker sequences translates at least to the order of 100 instances in the genome database. While many of those are plausible host protein interactions, others are less likely and may be random matches with regular expressions.

### Estimated accessible sequence and motif space of N-protein IDRs

Out of a total of 327 motif classes currently contained in the ELM database, the total number of motif classes displayed in N-protein sequences is 72 in the linker, 62 in the C-arm, and 53 in the N-arm IDR. This raises the question of how efficiently random mutations can create new motifs, and what fraction of the total currently known motif space is accessible to N-protein. Since the viable amino acid landscape (Fig. 1F) has been exhaustively explored during the pandemic so far, it is possible to make a back-of-the-envelope estimate of the theoretical maximal size of the associated sequence space by permutation through viable amino acid mutations at each position. For the N-arm (1:44)—the smallest of the N-protein IDRs—allowing for three mutations per sequence, which is close to the average of ≈2.8 observed in the existing database, there are ≈1.8 × 10^6^ different mutant sequence permutations. Although likely not all will be viable due to epistatic effects, this upper limit is more than three orders of magnitude larger than the 512 distinct N-arm sequences observed so far in the genomic database and far exceeds our capacity for computational determination of the associated motif space.

**Fig 5:**
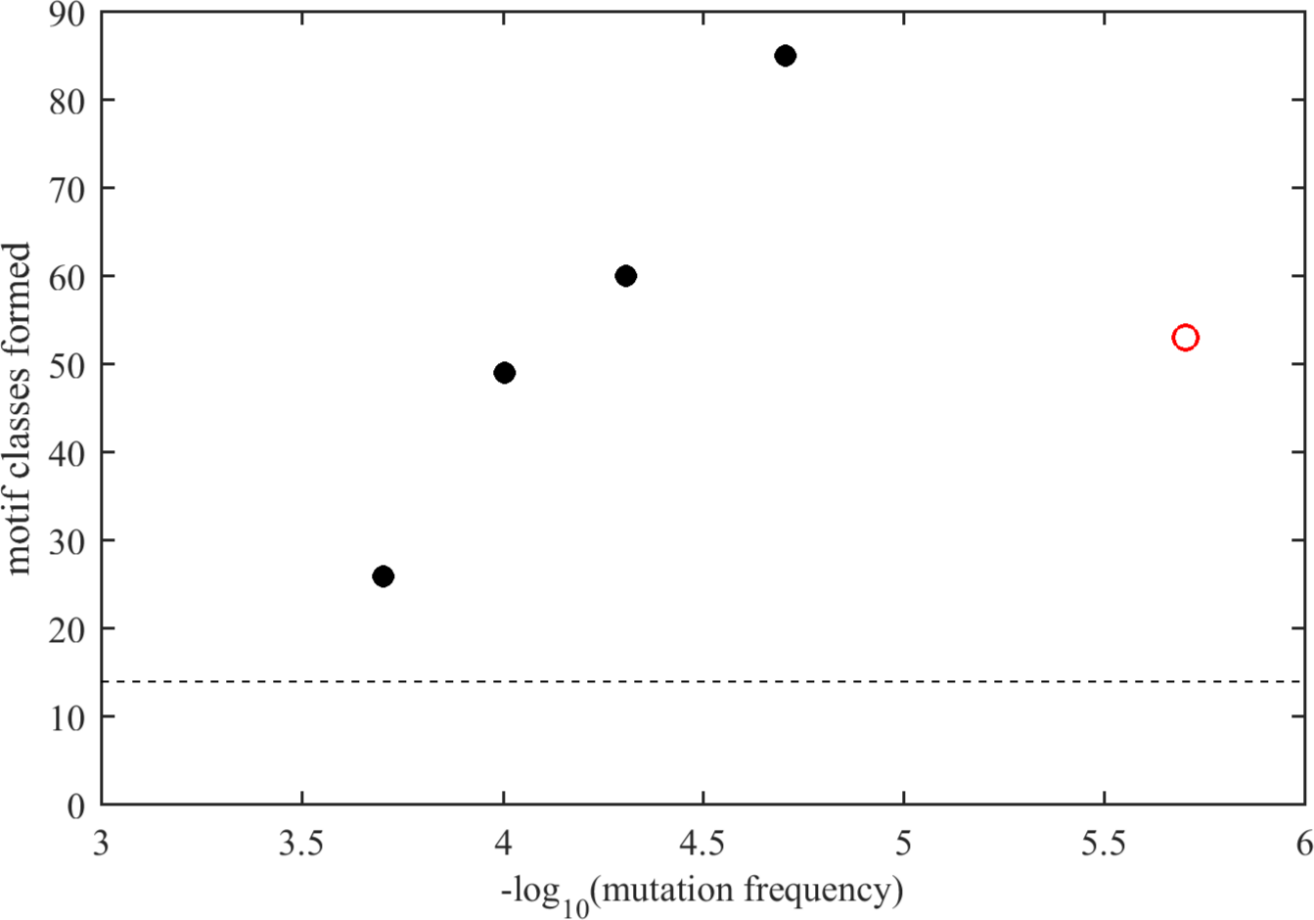
Estimated accessible motif space of N-protein N-arm IDR. Number of motif classes identified from the ELM database presented in the N-arm IDR examining a theoretical sequence space formed by three mutations per sequence permutated from the amino acid landscape (Fig. 1F), considering only amino acid replacements with minimum frequencies indicated in the abscissa (number of observed mutation instances relative to a total of 5.06 million sequences). Black circles are completely evaluated theoretical sequence spaces, and the red circle is based on 512 distinct N-arm sequences contained in the GISAID database, comprising ≈0.03% of the theoretical sequence space. The dashed horizontal line is the number of motif classes displayed in the ancestral N-arm sequence.

Nonetheless, limited approximate sequence spaces can be searched when focusing on only the most frequently encountered amino acid mutations. For example, considering only the mutations observed in >1,000 instances (i.e., in 0.02% of genomes; depicted in bold red in Fig. 1F), the associated sequence space with three mutations consists of 10,500 sequences, which is searchable and describes 26 motif classes from the ELM database, nearly twice the number of different motifs in the ancestral N-arm sequence. Lowering the mutation frequency threshold leads to a rapidly growing sequence space and associated motif space (Fig. 5). For example, at a mutation threshold of >100 instances (mutation frequency of 0.002% in all genomes), 231,519 possible sequences with three mutations cover a range of 85 motif classes or 26% of all in the ELM database. Consideration of more rare mutations occurring in the viable amino acid landscape further increases the theoretical sequence space and thereby the accessible motif space.

## DISCUSSION

The virus-host interface is crucial for viral survival and a promising area for the development of antiviral therapeutics. Significant recent advances were driven by increased understanding of the evolutionary role of IDRs and the recognition of SLiMs as ubiquitous interaction modules that can be hijacked through viral mimicry. Unavoidably, both large-scale experimental and bioinformatics studies contributing to this picture were limited to consensus sequences of viral species, lacking opportunity to account for the quasispecies nature of RNA viruses, and thus leaving the salient feature of sequence diversity previously unexplored with regard to the role of SLiMs in the virus-host interface. However, an important recent development is the assembly of a vast SARS-CoV-2 genomic repository at GISAID (30), which exceeds the number of available influenza sequences by more than one order of magnitude. This allows for the first time the exhaustive characterization of the amino acid mutation landscape (31–33) and is exploited here to power an analysis of the viral sequence space. This illuminates the highly dynamic motif space of viral IDRs, providing new insights in the unexpected efficiency of *ex nihilo* motif creation, the extent of viral motif mimicry, and the potential size of the virus-host interface.

We have started the present work with the assembly of the amino acid mutational landscapes from the host population-wide ensemble of consensus sequences. In a first approximation, we may consider the amino acid landscape as a reflection of all mutations consistent with vital biophysical, functional constraints of the viral protein, from which random sequence samples arise with certain mutation frequencies. Grouping all SARS-CoV-2 N-protein sequences in three major waves from different periods of the pandemic, groups essentially representing independent repeats of deep mutational scans, produces nearly identical mutational landscapes and local mutation frequencies. This suggests that the basic biophysical properties of N-protein overall have not significantly changed, as in an evolutionary stable steady state. In the first approximation, this view justifies considering the derived motif space to be similarly in steady state and to represent an intrinsic property of N-protein.

This is notwithstanding fitness modulations from localized N-protein such as 203K/204R (57) and 215C (31) that seem secondary to evolution of the immunodominant spike protein. Interestingly, in both Delta and Omicron waves, the defining mutations lead to the destruction of one PKA and one SRPK motif that may impact the extent of linker phosphorylation and modulate the switch between intracellular and assembly functions (57). On the other hand, the observed variation in the content of kinase motifs is very large, and none of the potential phosphorylation sites other than S184 is strongly conserved in the mutational landscape, apparently without compromising virus viability. This points to a distributed phosphorylation threshold in the linker IDR rather than specific structural requirements (66) for N-protein to be viable, which may be fine tuned for fitness optimization.

The data for the available time scale depict highly parallel random exploration of many motifs. On the level of single proteins, multiple overlapping repeat instances of motifs are displayed along the IDRs, a feature frequently encountered RNA viruses (14), which may lead to cooperativity and effective enhancement (64, 67). Similarly, different overlapping motifs provide multi-functionality with little competition due to the large expression level with 10^8^ copies of N-protein in the infected cells (35). Across the mutant spectrum, we observe highly effective motif creation, spanning an astonishingly wide range theoretically covering 20% or more of the known eukaryotic motif space (approximated as those contained in the ELM database) even in the shortest N-protein IDR. Conversely, most of the motifs displayed in the ancestral sequence are destroyed in at least a subset of the viable mutant spectrum.

The origin of the sequence diversity in the consensus sequences considered here is rooted in the error-prone transcription and the intracellular quasispecies. However, it is unclear to what extent the observed SARS-CoV-2 sequence space reflects intracellular quasispecies and intrahost “meta-quasispecies” (27), perhaps constituting a “hyper-quasispecies” as the ensemble of consensus sequences across the host population, with the obvious exclusion of non-viable species. Even though the amino acid mutation landscape (Fig. 1F) is sufficiently sampled to reflect biophysical features (36), only a very small fraction of the implied theoretical sequence space has been sampled. Even less is known about the quasispecies, with deep sequencing capabilities probing intrahost minority sequences currently limited to a level of 0.1% (68). However, several studies report that most of the mutations of intrahost minority species are independently reflected in the GISAID repository of consensus sequences (68–70), demonstrating at least a significant overlap. If the motif space observed in the present work is any indication of intracellular diversity, this would strongly further leverage the viral motif range. For example, it raises the possibility of cooperation between species (71), and one could envision minority species to interact with host proteins through motifs that the master sequence has abandoned. Thus, interaction motifs that disappear in a subset of consensus sequences in our study may nonetheless still be essential for viable virus. Further, the simultaneous presence of viral protein species with different motif sets attacking host processes at multiple entry points may exert synergistic effects on redundant interaction networks (72).

Whether the observed motif diversity extends intracellularly or not, the present work demonstrates that single sequence-based studies of viral protein/host interactions neglecting viral diversity likely vastly underestimate the abundance of SLiM-based protein-protein interactions in the virus-host interface. While many of the displayed motifs may not be functional host protein interactions, due to incorrect sequence context, localization, or even species specificity, the present study is also limited by the still incomplete and expanding number of SLiM classes. This does not diminish the role of the dynamics of random motif generation observed here, which appears to serve as a fertile basis to search for beneficial interactions for further optimization and host adaptation. Non-functional interactions as a consequence of “evolutionary noise” may have little fitness penalty and should be expected to occur, as pointed out by Levy and colleagues (73), and this should hold true in particular for viral quasispecies.

## MATERIALS AND METHODS

### Mutational landscape

Mutation data were based on consensus sequences of SARS-CoV-2 isolates submitted to GISAID and downloaded on 20 January 2023 as database files metadata.tsv and nextclade.tsv preprocessed by Nextstrain (61). These contained ≈7.23 million genomes, of which only 5.06 million high-quality sequences based on multiple criteria evaluated in the Nextstrain workflow were included here. As described previously (31), for inspection of the mutational landscape, a threshold of 10 observations of any mutation was used to filter adventitious sequencing errors. A total of 746 sequences exhibiting insertions in the N protein were omitted. The Wuhan-Hu-1 isolate (GenBank QHD43423) (74) was used as the ancestral reference. Alignment of SARS and related betacoronavirus sequences was carried out with COBALT at NLM (75). SARS-CoV-2 sequences were grouped in sets of Omicron variants, Delta variants (Nextstrain 21J and later Delta clades), and sequences preceding 21J including Alpha, Beta, and other variants as well as ancestral sequences (termed pre-Delta). Processing, plotting, and analysis of the sequence data were performed with MATLAB (Mathworks, Natick, MA).

### Prediction of N-protein disorder and visualization

The N-protein structure was predicted using ColabFold (76). Since no confidence is achieved for residue angles in disordered regions, these were artificially stretched closer to 180° for better visualization. The resulting structure was plotted using ChimeraX (77) and colored according to the predicted IUPred3 score (78).

### Analysis of SLiMs

SLiMs pattern recognition was carried out on distinct amino acid sequences with mutations in the disordered regions of interest, i.e., the N-arm (1–44) , linker (180–247), and C-arm (364–419). To this end, all 5.06 million sequences were classified according to their N-protein IDR amino acid sequences. Among the variant groups, a total of 611 distinct classes of N-arm sequences, 1045 distinct linker sequences, and 685 C-arm sequences were identified that occurred in more than a threshold of 10 genomes (or 502, 967, and 532 distinct sequences for N-arm, linker, and C-arm, respectively, discounting duplicates between variant groups).

In a preprocessing step, deletions were removed; sequence regions of interest were extended by 10 aa and, for efficiency to reduce server traffic, concatenated with AAAAA spacers to create composite sequences of length ≈1,000 aa prior to submission to the Eukaryotic Linear Motif Resource server (http://elm.eu.org) (11). This concatenation process excludes from the statistics all N- and C-terminal-specific SLiMs, such as LIG_BIR_II_1 at the N-terminus of the N-arm and LIG_PDZ_Class_1 at the C-terminus of the C-arm, but avoids creating artificial termini at the limits of the IDR sequences of interest. System CURL commands were issued from a MATLAB script to send and receive data, to carry out communication error control, and to parse the returned text to extract motif data. Motif content was mapped back and cropped onto the original sequence framework of interest, and motif name, multiplicity, and starting and ending positions were tabulated for statistical analysis. Motifs with alternate regular expressions that completely overlapped were counted as a single instance. The frequency distribution of motif multiplicities across the different IDR sequence classes was derived from the table of motif multiplicity in each sequence.

Since motifs of two known key N-protein interactions with host proteins are not part of the ELM database, the sequence analysis was appended by an offline search for the ϕ-x-F motif for N-protein binding of the NTF2-like domain of G3BP1 (51) and a search for the RSxSxSR and RxxSPxR motifs binding of the SRPK kinase (55). Their instances are labeled as “+G3 BP1+” and “+SRPK +,” respectively, with the deviation from the ELM database nomenclature indicating their origin from a separate search.

To assess the probability of any SLiM occurring by chance, motif searches on 10,000 random sequences matching the amino acid content of the ancestral sequence of disordered linker and arms, respectively, were carried out. This was accomplished by performing random permutations reassigning amino acids to random positions within each IDR. The resulting sequences were analyzed for their motif content using the same computational pipeline as described for the sequence data above.

The analysis of hypothetical sequence space was carried out for the N-arm on the basis of the ancestral Wuhan-Hu-1 sequence and the mutational landscape of observed amino acid mutations. In a first step, a set of possible mutations was created given a threshold of instances (or mutation frequency) for mutations to be considered. This creates an amino acid mutation table *a_pm_* of possible replacements of the ancestral residue by mutation *m* at position *p*, with 0 ≤ *m* ≤ *M_p_*, where *M_p_* is the total number of mutations above the threshold frequency *f* at that position *p*. In a second step, the set of possible sequences with three mutations was created in MATLAB by permutation through all combinations (*a_ix_*, *a_jy_, a_kz_*) with *i* < *j* < k with 1 ≤ *x* ≤ *M_i_*, 1 ≤ *y* ≤ *M_j_*, and 1 ≤ *z* ≤ *M_k_*, avoiding redundant symmetric permutations. The resulting amino acids at positions *i*, *j*, and *k* replaced the amino acids in the ancestral sequence. To determine the motif space associated with the accessible sequence space, each of the resulting sequences was subjected to the same motif analysis pipeline described above. This analysis was repeated for different threshold frequencies, which creates an extended set of considered amino acid mutations. For efficiency in the analysis of next lower threshold frequencies, only the new sequences not already contained in the previous set of higher threshold mutations were subjected to motif analysis, and results were merged with those already obtained at the higher threshold.

## Data Availability

A spreadsheet with the list of all SLiMs and their distribution in N-arm, linker, and C-arm IDRs of SARS-CoV-2 nucleocapsid protein can be downloaded from the Harvard Dataverse at https://doi.org/10.7910/DVN/HOKVVE.

## SUPPLEMENTAL MATERIAL

Figure S1 mbio.02388-23-s0001.tiff Mutational landscape of SARS-CoV-2 Omicron N-protein. As in Fig. 1, shown in bold black letters is the ancestral amino acid sequence (Wuhan-Hu-1), and on top are mutations found in 5.05 million sequences from the GISAID repository, downloaded from Nextstrain in January 2023, and classified as one of the Omicron clades. The mutations are ordered and colored by number of genomes found to contain that specific mutation: 10 to 100 (gray), 100 to 500 (blue), 500 to 1,000 (purple), and > 1,000 (red). The intrinsically disordered regions are highlighted: Narm in yellow, the linker in magenta, and C-arm in cyan.

ASM does not own the copyrights to Supplemental Material that may be linked to, or accessed through, an article. The authors have granted ASM a non-exclusive, world-wide license to publish the Supplemental Material files. Please contact the corresponding author directly for reuse.

## References

[1] Sangster AG, Zarin T, Moses AM. 2022. Evolution of short linear motifs and disordered proteins topic: yeast as model system to study evolution. Curr Opin Genet Dev 76:101964. doi:10.1016/j.gde.2022.101964 35939968

[2] Peti W, Page R. 2013. Molecular basis of MAP kinase regulation. Protein Sci 22:1698–1710. doi:10.1002/pro.2374 24115095 PMC3843625

[3] Tompa P, Davey NE, Gibson TJ, Babu MM. 2014. A million peptide motifs for the molecular biologist. Mol Cell 55:161–169. doi:10.1016/j.molcel.2014.05.032 25038412

[4] Wang X, Bajaj R, Bollen M, Peti W, Page R. 2016. Expanding the Pp2A interactome by defining a B56-specific slim. Structure 24:2174–2181. doi:10.1016/j.str.2016.09.010 27998540 PMC5180209

[5] Ragusa MJ, Dancheck B, Critton DA, Nairn AC, Page R, Peti W. 2010. Spinophilin directs protein phosphatase 1 specificity by blocking substrate binding sites. Nat Struct Mol Biol 17:459–464. doi:10.1038/nsmb.1786 20305656 PMC2924587

[6] Shi G, Song C, Torres Robles J, Salichos L, Lou HJ, Lam TT, Gerstein M, Turk BE. 2023. Proteome-wide screening for mitogen-activated protein kinase docking motifs and interactors. Sci Signal 16:eabm5518. doi:10.1126/scisignal.abm5518 36626580 PMC9995140

[7] Sorgeloos F, Peeters M, Hayashi Y, Borghese F, Capelli N, Drappier M, Cesaro T, Colau D, Stroobant V, Vertommen D, de Bodt G, Messe S, Forné I, Mueller-Planitz F, Collet J-F, Michiels T. 2022. A case of convergent evolution: several viral and bacterial pathogens hijack RSK kinases through a common linear motif. Proc Natl Acad Sci U S A 119:e2114647119. doi:10.1073/pnas.2114647119 35091472 PMC8812568

[8] Mohapatra T, Dixit M. 2022. IQ motif containing GTPase activating proteins (IQGAPs), A-kinase anchoring proteins (AKAPs) and kinase suppressor of ras proteins (KSRs) in scaffolding oncogenic pathways and their therapeutic potential. ACS Omega 7:45837–45848. doi:10.1021/acsomega.2c05505 36570181 PMC9773950

[9] Srivastava G, Choy MS, Bolik-Coulon N, Page R, Peti W. 2023. Inhibitor-3 inhibits protein phosphatase 1 via a metal binding dynamic protein-protein interaction. Nat Commun 14:1798. doi:10.1038/s41467-023-37372-5 37002212 PMC10066265

[10] Manna A, Zhao H, Wada J, Balagopalan L, Tagad HD, Appella E, Schuck P, Samelson LE. 2018. Cooperative assembly of a four-molecule signaling complex formed upon T cell antigen receptor activation. Proc Natl Acad Sci U S A 115:E11914–E11923. doi:10.1073/pnas.1817142115 30510001 PMC6304999

[11] Kumar M, Michael S, Alvarado-Valverde J, Mészáros B, Sámano-Sánchez H, Zeke A, Dobson L, Lazar T, Örd M, Nagpal A, Farahi N, Käser M, Kraleti R, Davey NE, Pancsa R, Chemes LB, Gibson TJ. 2022. The Eukaryotic linear motif resource: 2022 release. Nucleic Acids Res 50:D497–D508. doi:10.1093/nar/gkab975 34718738 PMC8728146

[12] Davey NE, Simonetti L, Ivarsson Y. 2023. The next wave of Interactomics: mapping the slim-based interactions of the intrinsically disordered proteome. Curr Opin Struct Biol 80:102593. doi:10.1016/j.sbi.2023.102593 37099901

[13] Hagai T, Azia A, Babu MM, Andino R. 2014. Use of host-like peptide motifs in viral proteins is a prevalent strategy in host-virus interactions. Cell Rep 7:1729–1739. doi:10.1016/j.celrep.2014.04.052 24882001 PMC4089993

[14] Mihalič F, Simonetti L, Giudice G, Sander MR, Lindqvist R, Peters MBA, Benz C, Kassa E, Badgujar D, Inturi R, Ali M, Krystkowiak I, Sayadi A, Andersson E, Aronsson H, Söderberg O, Dobritzsch D, Petsalaki E, Överby AK, Jemth P, Davey NE, Ivarsson Y. 2023. Large-scale phage-based screening reveals extensive pan-viral mimicry of host short linear motifs. Nat Commun 14:2409. doi:10.1038/s41467-023-38015-5 37100772 PMC10132805

[15] Davey NE, Travé G, Gibson TJ. 2011. How viruses hijack cell regulation. Trends Biochem Sci 36:159–169. doi:10.1016/j.tibs.2010.10.002 21146412

[16] Shuler G, Hagai T. 2022. Rapidly evolving viral motifs mostly target biophysically constrained binding pockets of host proteins. Cell Rep 40:111212. doi:10.1016/j.celrep.2022.111212 35977510

[17] Simonetti L, Nilsson J, McInerney G, Ivarsson Y, Davey NE. 2023. SLiM-binding pockets: an attractive target for broad-spectrum antivirals. Trends Biochem Sci 48:420–427. doi:10.1016/j.tibs.2022.12.004 36623987

[18] Fuxreiter M, Tompa P, Simon I. 2007. Local structural disorder imparts plasticity on linear motifs. Bioinformatics 23:950–956. doi:10.1093/bioinformatics/btm035 17387114

[19] Tokuriki N, Oldfield CJ, Uversky VN, Berezovsky IN, Tawfik DS. 2009. Do viral proteins possess unique biophysical features? Trends Biochem Sci 34:53–59. doi:10.1016/j.tibs.2008.10.009 19062293

[20] Brown CJ, Takayama S, Campen AM, Vise P, Marshall TW, Oldfield CJ, Williams CJ, Dunker AK. 2002. Evolutionary rate heterogeneity in proteins with long disordered regions. J Mol Evol 55:104–110. doi:10.1007/s00239-001-2309-6 12165847

[21] Davey NE, Cyert MS, Moses AM. 2015. Short linear motifs - Ex nihilo evolution of protein regulation short linear motifs - the unexplored frontier of the eukaryotic proteome. Cell Commun Signal 13:9–11. doi:10.1186/s12964-015-0120-z 26589632 PMC4654906

[22] Neduva V, Russell RB. 2005. Linear motifs: evolutionary interaction switches. FEBS Lett 579:3342–3345. doi:10.1016/j.febslet.2005.04.005 15943979

[23] Gitlin L, Hagai T, LaBarbera A, Solovey M, Andino R. 2014. Rapid evolution of virus sequences in intrinsically disordered protein regions. PLoS Pathog 10:e1004529. doi:10.1371/journal.ppat.1004529 25502394 PMC4263755

[24] Mosca R, Pache RA, Aloy P. 2012. The role of structural disorder in the rewiring of protein interactions through evolution. Mol Cell Proteomics 11:M111. doi:10.1074/mcp.M111.014969 PMC3394948 22389433

[25] Cordeddu V, Di Schiavi E, Pennacchio LA, Ma’ayan A, Sarkozy A, Fodale V, Cecchetti S, Cardinale A, Martin J, Schackwitz W, et al.. 2009. Mutation of Shoc2 promotes aberrant protein N-Myristoylation and causes Noonan-like syndrome with loose Anagen hair. Nat Genet 41:1022–1026. doi:10.1038/ng.425 19684605 PMC2765465

[26] Domingo E, Holland JJ. 1997. RNA virus mutations and fitness for survival. Annu Rev Microbiol 51:151–178. doi:10.1146/annurev.micro.51.1.151 9343347

[27] Domingo E. 2019. Virus as Populations. Academic Press.

[28] Lauring AS, Andino R. 2010. Quasispecies theory and the behavior of RNA viruses. PLoS Pathog 6:e1001005. doi:10.1371/journal.ppat.1001005 20661479 PMC2908548

[29] Eigen M. 1993. Viral quasispecies. Sci Am 269:42–49. doi:10.1038/scientificamerican0793-42 8337597

[30] Elbe S, Buckland-Merrett G. 2017. Data, disease and diplomacy: GISAID’s innovative contribution to global health. Glob Chall 1:33–46. doi:10.1002/gch2.1018 31565258 PMC6607375

[31] Zhao H, Nguyen A, Wu D, Li Y, Hassan SA, Chen J, Shroff H, Piszczek G, Schuck P. 2022. Plasticity in structure and assembly of SARS-CoV-2 nucleocapsid protein. PNAS Nexus 1:gac049. doi:10.1093/pnasnexus/pgac049 PMC9235412 35783502

[32] Bloom JD, Beichman AC, Neher RA, Harris K. 2023. Evolution of the SARS-CoV-2 mutational spectrum. Mol Biol Evol 40:msad085. doi:10.1093/molbev/msad085 37039557 PMC10124870

[33] Saldivar-Espinoza B, Garcia-Segura P, Novau-Ferré N, Macip G, Martínez R, Puigbò P, Cereto-Massagué A, Pujadas G, Garcia-Vallve S. 2023. The mutational landscape of SARS-CoV-2. Int J Mol Sci 24:9072. doi:10.3390/ijms24109072 37240420 PMC10219494

[34] Mészáros B, Sámano-Sánchez H, Alvarado-Valverde J, Čalyševa J, Martínez-Pérez E, Alves R, Shields DC, Kumar M, Rippmann F, Chemes LB, Gibson TJ. 2021. Short linear motif candidates in the cell entry system used by SARS-CoV-2 and their potential therapeutic implications. Sci Signal 14:eabd0334. doi:10.1126/scisignal.abd0334 33436497 PMC7928535

[35] Tugaeva KV, Hawkins D, Smith JLR, Bayfield OW, Ker D-S, Sysoev AA, Klychnikov OI, Antson AA, Sluchanko NN. 2021. The mechanism of SARS-CoV-2 nucleocapsid protein recognition by the human 14-3-3 proteins. J Mol Biol 433:166875. doi:10.1016/j.jmb.2021.166875 33556408 PMC7863765

[36] Zhao H, Wu D, Hassan SA, Nguyen A, Chen J, Piszczek G, Schuck P. 2023. A conserved oligomerization domain in the disordered Linker of coronavirus nucleocapsid proteins. Sci Adv 9:eadg6473. doi:10.1126/sciadv.adg6473 37018390 PMC10075959

[37] Carlson CR, Adly AN, Bi M, Howard CJ, Frost A, Cheng Y, Morgan DO. 2022. Reconstitution of the SARS-CoV-2 ribonucleosome provides insights into genomic RNA packaging and regulation by phosphorylation. J Biol Chem 298:102560. doi:10.1016/j.jbc.2022.102560 36202211 PMC9529352

[38] Masters PS. 2019. Coronavirus genomic RNA packaging. Virology 537:198–207. doi:10.1016/j.virol.2019.08.031 31505321 PMC7112113

[39] Gordon DE, Jang GM, Bouhaddou M, Xu J, Obernier K, White KM, O’Meara MJ, Rezelj VV, Guo JZ, Swaney DL, et al.. 2020. A SARS-CoV-2 protein interaction map reveals targets for drug repurposing. Nature 583:459–468. doi:10.1038/s41586-020-2286-9 32353859 PMC7431030

[40] Kruse T, Benz C, Garvanska DH, Lindqvist R, Mihalic F, Coscia F, Inturi R, Sayadi A, Simonetti L, Nilsson E, Ali M, Kliche J, Moliner Morro A, Mund A, Andersson E, McInerney G, Mann M, Jemth P, Davey NE, Överby AK, Nilsson J, Ivarsson Y. 2021. Large scale discovery of coronavirus-host factor protein interaction motifs reveals SARS-CoV-2 specific mechanisms and vulnerabilities. Nat Commun 12:6761. doi:10.1038/s41467-021-26498-z 34799561 PMC8605023

[41] Zheng X, Sun Z, Yu L, Shi D, Zhu M, Yao H, Li L. 2021. Interactome analysis of the nucleocapsid protein of SARS-CoV-2 virus. Pathogens 10:1155. doi:10.3390/pathogens10091155 34578187 PMC8465953

[42] Wu W, Cheng Y, Zhou H, Sun C, Zhang S. 2023. The SARS-CoV-2 nucleocapsid protein: Its role in the viral life cycle, structure and functions, and use as a potential target in the development of vaccines and diagnostics. Virol J 20:6. doi:10.1186/s12985-023-01968-6 36627683 PMC9831023

[43] Li J-Y, Liao C-H, Wang Q, Tan Y-J, Luo R, Qiu Y, Ge X-Y. 2020. The ORF6, ORF8 and nucleocapsid proteins of SARS-CoV-2 inhibit type I interferon signaling pathway. Virus Res 286:198074. doi:10.1016/j.virusres.2020.198074 32589897 PMC7309931

[44] Mu J, Fang Y, Yang Q, Shu T, Wang A, Huang M, Jin L, Deng F, Qiu Y, Zhou X. 2020. SARS-CoV-2 N protein antagonizes type I interferon signaling by suppressing phosphorylation and nuclear translocation of STAT1 and STAT2. Cell Discov 6:65. doi:10.1038/s41421-020-00208-3 32953130 PMC7490572

[45] Yelemali P, Hao L, Liu Q. 2022. Mechanisms of host type I interferon response modulation by the nucleocapsid proteins of alpha- and betacoronaviruses. Arch Virol 167:1925–1930. doi:10.1007/s00705-022-05513-8 35763067 PMC9244355

[46] Pan P, Shen M, Yu Z, Ge W, Chen K, Tian M, Xiao F, Wang Z, Wang J, Jia Y, Wang W, Wan P, Zhang J, Chen W, Lei Z, Chen X, Luo Z, Zhang Q, Xu M, Li G, Li Y, Wu J. 2021. SARS-CoV-2 N protein promotes NLRP3 Inflammasome activation to induce hyperinflammation. Nat Commun 12:4664. doi:10.1038/s41467-021-25015-6 34341353 PMC8329225

[47] Gao T, Zhu L, Liu H, Zhang X, Wang T, Fu Y, Li H, Dong Q, Hu Y, Zhang Z, et al.. 2022. Highly pathogenic coronavirus N protein aggravates inflammation by MASP-2-mediated lectin complement pathway overactivation. Signal Transduct Target Ther 7:318. doi:10.1038/s41392-022-01133-5 36100602 PMC9470675

[48] Yuan S, Yan B, Cao J, Ye Z-W, Liang R, Tang K, Luo C, Cai J, Chu H, Chung T-H, To K-W, Hung I-N, Jin D-Y, Chan J-W, Yuen K-Y. 2021. SARS-CoV-2 exploits host DGAT and ADRP for efficient replication. Cell Discov 7:100. doi:10.1038/s41421-021-00338-2 34702802 PMC8548329

[49] Karwaciak I, Sałkowska A, Karaś K, Dastych J, Ratajewski M. 2021. Nucleocapsid and spike proteins of the coronavirus SARS-CoV-2 induce IL6 in monocytes and macrophages—potential implications for cytokine storm syndrome. Vaccines (Basel) 9:54. doi:10.3390/vaccines9010054 33467724 PMC7830532

[50] López-Muñoz AD, Kosik I, Holly J, Yewdell JW. 2022. Cell surface SARS-CoV-2 nucleocapsid protein modulates innate and adaptive immunity. Sci Adv 8:eabp9770. doi:10.1126/sciadv.abp9770 35921414 PMC9348789

[51] Biswal M, Lu J, Song J. 2022. SARS-CoV-2 nucleocapsid protein targets a conserved surface groove of the NTF2-like domain of G3BP1. J Mol Biol 434:167516. doi:10.1016/j.jmb.2022.167516 35240128 PMC8882607

[52] Yang Z, Johnson BA, Meliopoulos VA, Ju X, Zhang P, Hughes MP, Wu J, Koreski KP, Chang T-C, Wu G, et al.. 2023. Interaction between host G3BP and viral nucleocapsid protein regulates SARS-CoV-2 replication. bioRxiv:2023.06.29.546885. doi:10.1101/2023.06.29.546885 PMC11044841 38492217

[53] Eisenreichova A, Boura E. 2022. Structural basis for SARS-CoV-2 nucleocapsid (N) protein recognition by 14-3-3 proteins. J Struct Biol 214:107879. doi:10.1016/j.jsb.2022.107879 35781025 PMC9245327

[54] Tugaeva KV, Sysoev AA, Kapitonova AA, Smith JLR, Zhu P, Cooley RB, Antson AA, Sluchanko NN. 2023. Human 14-3-3 proteins site-selectively bind the mutational hotspot region of SARS-CoV-2 nucleoprotein modulating its phosphoregulation. J Mol Biol 435:167891. doi:10.1016/j.jmb.2022.167891 36427566 PMC9683861

[55] Yaron TM, Heaton BE, Levy TM, Johnson JL, Jordan TX, Cohen BM, Kerelsky A, Lin T-Y, Liberatore KM, Bulaon DK, et al.. 2022. Host protein kinases required for SARS-CoV-2 nucleocapsid phosphorylation and viral replication. Sci Signal 15:eabm0808. doi:10.1126/scisignal.abm0808 36282911 PMC9830954

[56] Carlson CR, Asfaha JB, Ghent CM, Howard CJ, Hartooni N, Safari M, Frankel AD, Morgan DO. 2020. Phosphoregulation of phase separation by the SARS-CoV-2 N protein suggests a biophysical basis for its dual functions. Mol Cell 80:1092–1103. doi:10.1016/j.molcel.2020.11.025 33248025 PMC7677695

[57] Syed AM, Ciling A, Chen IP, Carlson CR, Adly A, Martin H, Taha TY, Khalid MM, Bouhaddou M, Ummadi MR, Moen JM, Krogan NJ, Morgan DO, Ott M, Doudna J. 2023. SARS-CoV-2 evolution balances conflicting roles of N protein phosphorylation. SSRN. doi:10.2139/ssrn.4472729 PMC11620656 39571001

[58] Fung TS, Liu DX. 2018. Post-translational modifications of coronavirus proteins: roles and function. Future Virol 13:405–430. doi:10.2217/fvl-2018-0008 32201497 PMC7080180

[59] Mao S, Cai X, Niu S, Wei J, Jiang N, Deng H, Wang W, Zhang J, Shen S, Ma Y, Wu X, Peng Q, Huang A, Wang D. 2023. TRIM21 promotes ubiquitination of SARS‐CoV‐2 nucleocapsid protein to regulate innate immunity. J Med Virol 95:e28719. doi:10.1002/jmv.28719 37185839

[60] Madahar V, Dang R, Zhang Q, Liu C, Rodgers VGJ, Liao J. 2023. Human post-translational sumoylation modification of SARS-CoV-2 nucleocapsid protein enhances its interaction affinity with itself and plays a critical role in its nuclear translocation. Viruses 15:1600. doi:10.3390/v15071600 37515286 PMC10384427

[61] Hadfield J, Megill C, Bell SM, Huddleston J, Potter B, Callender C, Sagulenko P, Bedford T, Neher RA. 2018. Nextstrain: real-time tracking of pathogen evolution. Bioinformatics 34:4121–4123. doi:10.1093/bioinformatics/bty407 29790939 PMC6247931

[62] Bloom JD, Neher RA. 2023. Fitness effects of mutations to SARS-CoV-2 proteins. Virus Evol 9:vead055. doi:10.1093/ve/vead055 37727875 PMC10506532

[63] Liu X, Verma A, Garcia G, Ramage H, Lucas A, Myers RL, Michaelson JJ, Coryell W, Kumar A, Charney AW, Kazanietz MG, Rader DJ, Ritchie MD, Berrettini WH, Schultz DC, Cherry S, Damoiseaux R, Arumugaswami V, Klein PS. 2021. Targeting the coronavirus nucleocapsid protein through GSK-3 inhibition. Proc Natl Acad Sci U S A 118:1–9. doi:10.1073/pnas.2113401118 PMC8594528 34593624

[64] Klein P, Pawson T, Tyers M. 2003. Mathematical modeling suggests cooperative interactions between a disordered polyvalent ligand and a single receptor site. Curr Biol 13:1669–1678. doi:10.1016/j.cub.2003.09.027 14521832

[65] Shajahan A, Pepi LE, Rouhani DS, Heiss C, Azadi P. 2021. Glycosylation of SARS-CoV-2: structural and functional insights. Anal Bioanal Chem 413:7179–7193. doi:10.1007/s00216-021-03499-x 34235568 PMC8262766

[66] Zarin T, Strome B, Peng G, Pritišanac I, Forman-Kay JD, Moses AM. 2021. Identifying molecular features that are associated with biological function of intrinsically disordered protein regions. Elife 10:e60220. doi:10.7554/eLife.60220 33616531 PMC7932695

[67] Watson M, Almeida TB, Ray A, Hanack C, Elston R, Btesh J, McNaughton PA, Stott K. 2022. Hidden multivalency in phosphatase recruitment by a disordered AKAP scaffold. J Mol Biol 434:167682. doi:10.1016/j.jmb.2022.167682 35697294

[68] Martínez-González B, Soria ME, Vázquez-Sirvent L, Ferrer-Orta C, Lobo-Vega R, Mínguez P, de la Fuente L, Llorens C, Soriano B, Ramos-Ruíz R, et al.. 2022. SARS-CoV-2 mutant spectra at different depth levels reveal an overwhelming abundance of low frequency mutations. Pathogens 11:662. doi:10.3390/pathogens11060662 35745516 PMC9227345

[69] Tonkin-Hill G, Martincorena I, Amato R, Lawson ARJ, Gerstung M, Johnston I, Jackson DK, Park N, Lensing SV, Quail MA, et al.. 2021. Patterns of within-host genetic diversity in SARS-CoV-2. Elife 10:e66857. doi:10.7554/eLife.66857 34387545 PMC8363274

[70] Siqueira JD, Goes LR, Alves BM, de Carvalho PS, Cicala C, Arthos J, Viola JPB, de Melo AC, Soares MA. 2021. SARS-CoV-2 genomic analyses in cancer patients reveal elevated intrahost genetic diversity. Virus Evol 7:veab013. doi:10.1093/ve/veab013 33738124 PMC7928633

[71] Vignuzzi M, Stone JK, Arnold JJ, Cameron CE, Andino R. 2006. Quasispecies diversity determines pathogenesis through cooperative interactions in a viral population. Nature 439:344–348. doi:10.1038/nature04388 16327776 PMC1569948

[72] Levy ED, Landry CR, Michnick SW. 2010. Cell signaling. Signaling through cooperation. Science 328:983–984. doi:10.1126/science.1190993 20489011

[73] Levy ED, Landry CR, Michnick SW. 2009. How perfect can protein Interactomes be? Sci Signal 2:pe11. doi:10.1126/scisignal.260pe11 19261595

[74] Wu F, Zhao S, Yu B, Chen Y-M, Wang W, Song Z-G, Hu Y, Tao Z-W, Tian J-H, Pei Y-Y, Yuan M-L, Zhang Y-L, Dai F-H, Liu Y, Wang Q-M, Zheng J-J, Xu L, Holmes EC, Zhang Y-Z. 2020. A new Coronavirus associated with human respiratory disease in China. Nature 579:265–269. doi:10.1038/s41586-020-2202-3 32015508 PMC7094943

[75] Papadopoulos JS, Agarwala R. 2007. COBALT: constraint-based alignment tool for multiple protein sequences. Bioinformatics 23:1073–1079. doi:10.1093/bioinformatics/btm076 17332019

[76] Mirdita M, Schütze K, Moriwaki Y, Heo L, Ovchinnikov S, Steinegger M. 2022. Colabfold: making protein folding accessible to all. Nat Methods 19:679–682. doi:10.1038/s41592-022-01488-1 35637307 PMC9184281

[77] Pettersen EF, Goddard TD, Huang CC, Meng EC, Couch GS, Croll TI, Morris JH, Ferrin TE. 2021. UCSF chimerax: structure visualization for researchers, educators, and developers. Protein Sci 30:70–82. doi:10.1002/pro.3943 32881101 PMC7737788

[78] Erdős G, Pajkos M, Dosztányi Z. 2021. IUPred3: prediction of protein disorder enhanced with unambiguous experimental annotation and visualization of evolutionary conservation. Nucleic Acids Res 49:W297–W303. doi:10.1093/nar/gkab408 34048569 PMC8262696

